# Anticipated resource utilization for injury versus non-injury pediatric visits to emergency departments

**DOI:** 10.1186/s40621-016-0077-4

**Published:** 2016-05-03

**Authors:** Mark R. Zonfrillo, Michelle L. Macy, Lawrence J. Cook, Tomohiko Funai, Rachel M. Stanley, James M. Chamberlain, Rebecca M. Cunningham, Elizabeth R. Alpern, For the Pediatric Emergency Care Applied Research Network (PECARN)

**Affiliations:** 1 Department of Emergency Medicine, Alpert Medical School of Brown University, 55 Claverick Street, 2nd Floor, Providence, RI 02903 USA; 2 Injury Prevention Center, Hasbro Children’s Hospital, Providence, RI USA; 3 Department of Emergency Medicine, University of Michigan, Ann Arbor, MI USA; 4 University of Michigan Injury Center, Ann Arbor, MI USA; 5 Child Health Evaluation and Research (CHEAR) Unit, Division of General Pediatrics, University of Michigan, Ann Arbor, MI USA; 6 Department of Pediatrics, University of Utah, Salt Lake City, UT USA; 7 Division of Emergency Medicine, Nationwide Children’s Hospital, Columbus, OH USA; 8 Department of Pediatrics & Emergency Medicine, Children’s National Health System, George Washington University, Washington, DC, USA; 9 Ann and Robert H. Lurie Children’s Hospital of Chicago, Feinberg School of Medicine, Northwestern University, Chicago, IL USA

**Keywords:** Injury severity, Resource utilization, Emergency department, Child, Adolescent, Coding systems

## Abstract

**Background:**

Childhood injuries are increasingly treated in emergency departments (EDs) but the relationship between injury severity and ED resource utilization has not been evaluated. The objective of this study was to compare resource utilization for pediatric injury-related ED visits across injury-severity levels and with non-injury visits, using standardized, validated scales.

**Methods:**

A retrospective analysis of 2004-2008 ED visits from the Pediatric Emergency Care Applied Research Network Core Data Project. Maximum Abbreviated Injury Scale severity (MAIS) and Severity Classification System (SCS) scores were calculated and compared. MAIS and SCS are ordinal scales from 1 (minor injury) to 6, and 1 (low anticipated resource utilization) to 5, respectively. ED length of stay (LOS) and admission percentages were calculated as comparative proxy measures of resource utilization.

**Results:**

There were 763,733 injury visits and 2,328,916 non-injury visits, most with SCS of 2 or 3. Of the injured patients, 59.2 % had an MAIS of 1. ED LOS and admission percentage increased with increasing MAIS from 1-5. LOS and admission percentage increased with increasing SCS in both samples. Median LOS was shorter for injured versus non-injured patients with SCS 3-5. Non-injured patients with SCS 2-5 were more likely admitted than injured patients. Most injured patients had an SCS 3 with an MAIS 1-2, or an SCS 2 with an MAIS 1, with no correlation between the two scales.

**Conclusion:**

While admission rates and LOS increase with increasing AIS and SCS severity, these two classification schemas do not reliably correlate. Similarly, ED visit metrics differ between injured and non-injured patients in similar SCS categories. Although AIS and SCS both have value, these differences should be considered when using these schemas in research and quality improvement.

## Background

Injury is the leading cause of mortality and acquired disability in children, (Centers for Disease Control and Prevention 2010) and it is estimated that for every fatal injury there are 25 children hospitalized and 925 treated in the emergency department (ED). (Centers for Disease Control and Prevention 2012) Although fatal childhood injuries have decreased over time, (Centers for Disease Control and Prevention 2012) ED visits for injuries in children have increased. (Thompson et al. 2012; Sharpe et al. 2012; Harris et al. 2011; Howell et al. 2010; D'Ippolito et al. 2010).

We recently described pediatric injury-related ED visits over 5 years from 2004-2008, including individual- and community-level socio-demographic characteristics, and found an increase in ED visits over the study period but with a stable degree of injury severity. (Macy et al. 2015) Although we have evidence of increasing numbers of ED visits by children with low to moderate injury severity, the relationship between measures of injury severity and resource utilization for ED visits by children has had limited exploration. This is important since understanding patterns of anticipated resource needs could have implications for triaging and cohorting of patients in the ED setting.

The goals of this study were to 1) compare resource utilization for pediatric injury-related ED visits versus non-injury visits and 2) compare resource utilization across levels of injury severity using standardized, validated scales.

## Methods

### Data source

Investigators obtained data for this retrospective multicenter, multiyear study from the Pediatric Emergency Care Applied Research Network (PECARN) Core Data Project (PCDP), a database established from participating hospitals’ administrative and/or electronic health record data.(Alpern et al. 2006) We conducted analyses using data from 2004 through 2008. The institutional review boards of all participating sites and the data-coordinating center approved this study.

### Hospital selection

We considered sites eligible for the study based on three criteria: 1) the contribution of data for all study years and 2) the assignment of International Statistical Classification of Diseases 9^th^ Revision Clinical Modification (ICD-9-CM) E-codes to ≥80 % of visits with associated ICD-9-CM codes for injury (800 to 959.9).

### Setting

Of the 24 EDs contributing data to the PCDP continuously from 2004 to 2008, 16 were included in the analyses. Eight sites were freestanding children’s hospital EDs, 6 sites were separate pediatric EDs within a general ED, and 2 sites were general EDs. Annual pediatric visit numbers ranged from 10,437 to 84,301, with overall admission rates ranging from 3 to 22 %. Twelve of 16 sites were recognized as Level 1 Pediatric Trauma centers either by the American College of Surgeons and/or state or regional designation. Sites were located in the Northeast (*n* = 3), Mid-Atlantic (*n* = 5), Midwest (*n* = 6), and West (*n* = 2).

### Visit identification

ED visits by children <19 years of age were eligible for analysis. We categorized visits as injury-related based on: 1) the presence of an ICD-9-CM code for injury (800-959.9) in the first three diagnoses OR 2) the presence of any non-location E-code (E849.x). The latter criterion was used in order to capture visits with documented injury mechanisms, but without an injury code assigned. For example, a visit with an ICD-9-CM code for limb pain (729.5) AND an E-Code for motor vehicle collision (E813.x) was considered injury-related. Visits were considered non-injury-related if: all of the diagnosis codes were outside of 800-959.9 AND there was no injury mechanism E-code OR the only E-codes were considered “adverse effects” (E870-E879.99, E930-E949.99).

### Variables

Hospital characteristics included ED type (ED in a freestanding children’s hospital, pediatric ED within a general hospital, or general ED) and designation as a Level 1 Pediatric Trauma Center. Visit-level data elements within the PCDP included patient demographic characteristics (e.g., age, gender, race, payer), ICD-9-CM codes and E-codes, and outcome of the visit (admitted, transferred, ED death, or discharge/other). Child age was categorized according to groupings used by the CDC; <1 year, 1-4 years, 5-9 years, 10-14 years, 15-18 years (Centers for Disease Control and Prevention 2010).

### Maximum abbreviated injury scale (MAIS) severity

The AIS is anatomically based, consensus-derived, and is considered the global system of choice for injury severity determination. (Association for the Advancement of Automotive Medicine 2008) MAIS scores were calculated using a two-step process. First, an investigator certified in Abbreviated Injury Scale (AIS) scoring mapped each ICD-9-CM code associated with the visit to the 1998 version of the AIS (AIS98) codes using the ICDMAP-90 software, (MacKenzie et al. 1989) then manually re-mapped codes to the most recent AIS 2005/2008 versions using the AIS manual and the ICD-9-CM injury descriptions, (MacKenzie et al. 1989; Durbin et al. 2001; Association for the Advancement of Automotive Medicine 2008) which yielded the maximum AIS (MAIS) severity score value for each visit. This remapping was necessary to ensure the severities from the most recent manual were used. AIS severity scores are on an ordinal scale and range from 1 (minor) to 6 (untreatable). MAIS 2-6 injuries indicated moderate or more severe injuries. ICD-9-CM codes with insufficient detail to be mapped (e.g., 854, “Intracranial injury of other and unspecified nature”) and visits categorized as injury related based on an E-code without an ICD-9-CM code in the 800-959.9 range could not be mapped to an MAIS. These visits were determined as “un-mapped” and analyzed separately. Injury Severity Scores (ISS) were calculated by summing the squares of squares of the AIS scores of the three most severely injured body regions, and an MAIS 6 in any body region was assigned an ISS value of 75 (Association for the Advancement of Automotive Medicine 2008).

### Severity classification system (SCS)

*SCS scores* were used as one measure of the anticipated resource utilization for the visit. The SCS is a 5-level system in which ICD-9-CM diagnosis codes have been assigned a score related to the anticipated ED resource utilization for the care of a child with that diagnosis, which can be used to compare across any ED diagnosis (Alessandrini et al. 2012) SCS has been previously used to classify severity for various analyses of hospital visits. (Macy et al. 2012; Montalbano et al. 2016; Alpern et al. 2014) For the current study, each case was assigned the maximal SCS score among all ICD-9-CM codes associated with the visit. Within the SCS, a score of 1 indicates minor illness (e.g., diaper dermatitis) and 5 indicates major illness (e.g., septic shock). Each visit was assigned the maximal SCS category based on the highest SCS score among all ICD-9-CM codes associated with the visit.

### ED Visit characteristics

Additional measures of resource utilization included length of stay (LOS) and ED visit outcome. LOS was defined as the time between ED triage date/time and ED disposition date/time. ED visit outcome was categorized as admitted, transferred, ED death, or discharged/other (including left against medical advice, left without treatment, and missing).

### Analyses

Descriptive statistics were used to compare ED resource utilization (ED length of stay, discharge status) by SCS and MAIS level. Differences between the distribution of visit type (i.e., injury vs. non-injury) were compared using a chi-square test. Across the MAIS and SCS categories, differences in LOS were assessed using the Kruskal-Wallis test, increasing trend in median LOS was assessed using the likelihood ratio test, and differences in admission percentages were assessed using the Cochran-Armitage trend test. Single variable logistic regression was used to test for differences in the odds of admission/transfer/death between the injury and non-injury visits. Single variable quantile regression was used to compare the median length of stay by visit type. A scatter plot was created to examine the relationship between SCS and MAIS. Jittering was employed to better display the clustering of observations among specific combinations of SCS and MAIS.

## Results

There were 763,733 injury visits and 2,328,916 non-injury visits during the study period. Table 1 shows the patient and hospital characteristics of the sample. Approximately 2/3 of the sample (59.2 %) had an MAIS of 1. SCS 2 and 3 comprised the majority of injured and non-injured patients, although injured patients had a greater SCS 3 percentage.

**Table 1:** Patient and hospital level characteristics of study sample

	Injury visits *n* = 763,733	Non-Injury visits *n* = 2,328,916	All visits *n* = 3,092,649
	*n* (%)	*n* (%)	*n* (%)
Patient Characteristics			
Age, years			
< 1	40,574 (5.3)	505,622 (21.7)	546,196 (17.7)
1-4	234,000 (30.6)	834,789 (35.8)	1,068,789 (34.6)
5-9	175,982 (23.0)	424,685 (18.2)	600,667 (19.4)
10-14	188,651 (24.7)	307,596 (13.2)	496,247 (16.1)
15-18	124,526 (16.3)	256,224 (11.0)	380,750 (12.3)
Gender			
Male	450,287 (59.0)	1,209,644 (51.9)	1,659,931 (53.7)
Female	313,383 (41.0)	1,119,126 (48.1)	1,432,509 (46.3)
Unknown	63 (<0.1)	146 (<0.1)	209 (<0.1)
Race/Ethnicity			
White, non-Hispanic	299,494 (39.2)	670,458 (28.8)	969,952 (31.4)
Black, non-Hispanic	222,482 (29.7)	766,999 (32.9)	993,481 (32.1)
Hispanic	75,466 (9.9)	364,519 (15.7)	439,985 (14.2)
Other Race, non-Hispanic	38,724 (5.1)	121,620 (5.2)	160,344 (5.2)
Unknown	123,567 (16.2)	405,320 (17.4)	528,887 (17.1)
Payer Type			
Private	342,664 (44.9)	755,518 (32.4)	1,098,182 (35.5)
Public	355,754 (46.6)	1,384,977 (59.5)	1,740,731 (56.3)
Uninsured/Other	57,731 (7.6)	146,447 (6.3)	204,178 (6.6)
Unknown	7,584 (1.0)	41,974 (1.8)	49,558 (1.6)
Hospital Type			
Pediatric ED/Children’s Hospital	499,460 (65.4)	1,664,670 (71.5)	2,164,130 (70.0)
Pediatric ED within General ED	194,491 (25.5)	525,381 (22.6)	719,872 (23.3)
General ED	69,782 (9.1)	138,865 (6.0)	208,647 (6.8)
Level 1 Pediatric Trauma Center			
Yes	641,043 (83.9)	2,030,519 (87.2)	2,671,562 (86.4)
No	122,690 (16.1)	298,397 (12.8)	421,087 (13.6)

Table 2 shows the ED stay characteristics based on MAIS score for injury visits. ED LOS and admission percentage increased with increasing MAIS from 1-5 (2.6 % to 90.0 % respectively), and then decreased for MAIS 6 (42.9 %). Similarly, the largest proportion of fatal injuries had an MAIS of 6 (18.4 %). Median ISS reflected the MAIS categories, with most patients having only one ICD-9-CM injury code for their visit.

**Table 2:** ED stay characteristics based on Maximum Abbreviated Injury Scale (MAIS) score

Total injury visits	ED stay characteristic	Visits without MAIS mapping determine		MAIS^a^ 1	MAIS 2	MAIS 3	MAIS 4	MAIS 5	MAIS 6	Statistic^b^
		E-code without injury ICD-9	ICD-9 with insufficient detail to be mapped							
		*n* = 65,176	*n* = 109,195	*n* = 451,753	*n* = 120,183	*n* = 16,227	*n* = 914	*n* = 236	*n* = 49	
*N* = 763,733	% of all patients	8.5	14.3	59.2	15.7	2.1	0.1	0.03	0.01	---------
	LOS (mean)^c^	213.6	153.2	149.0	230.1	423.7	459.1	554.4	374.8	<0.001
	LOS (median)^d^	138.0	113.0	120.0	166.0	232.0	176.5	204.0	151.5	<0.001
	% admitted	14.2	5.9	2.6	17.6	79.2	89.2	90.0	42.9	<0.001
	% transferred	1.4	0.2	0.2	0.6	0.9	1.0	1.7	2.0	<0.001
	% died in ED	0.2	0.1	<0.1	<0.1	0.2	0.6	0.9	18.4	<0.001
	Median ISS	N/A	N/A	1	4	9	17	26	75	<0.001

^a^Maximum Abbreviated Injury Scale (AIS) score

^b^Testing differences in LOS (Kruskal-Wallis test), increasing trend in median LOS (likelihood ratio test), and Admit % across various MAIS (1-6 only) (Cochran-Armitage trend test)

^c^Corresponding mean Length of stay (LOS) for each MAIS in minutes (excluding visits resulting in death in ED *n* = 393)

^d^Corresponding median Length of stay (LOS) for each MAIS in minutes (excluding visits resulting in death in ED *n* = 393)

Tables 3 and 4 show the ED visit characteristics based on SCS for injured and non-injured patients, while Table 5 compares the characteristics for the two samples. ED LOS and admission rates increased with increasing SCS in both samples. Quantile regression demonstrated injured patients had shorter median LOS compared to non-injured patients for SCS 3-5 (37, 60, and 60 h respectively). Logistic regression showed that there was a modest but statistically significant decrease in the odds of injured patients being admitted for SCS categories 2-5 (OR = 0.79 to 0.62 for SCS = 2 and 5 respectively). The median ISS was 1 for SCS categories 1-3, with a slight increase to a median ISS of 3 and 10 for SCS 4 and 5, respectively.

**Table 3:** ED stay characteristics based on maximum Severity Classification Score (SCS) for injury visits

Total injury visits	ED stay characteristic	SCS	SCS^b^	SCS	SCS	SCS	SCS	Statistic^c^
		uncodable^a^	1	2	3	4	5	
		*N* = 8,632	*N* = 8,749	*N* = 218,681	*N* = 427,752	*N* = 73,329	*N* = 6,197	
*N* = 763,733	% of all patients	3.8	1.2	28.6	56.0	9.6	0.8	---------
	LOS (mean)^d^	191.3	109.8	138.3	170.9	284.8	386.6	<0.001
	LOS (median)^e^	123	75	112	121	180	180	<0.001
	% admit/ transfer/ died %^f^	7.5	1.1	1.5	6.7	36.3	73.4	<0.001
	Median ISS	1	1	1	1	2	10	<0.001

^a^ICD-9CM code could not be mapped to SCS

^b^Severity Classification System (SCS) for all diagnoses for visit

^c^Testing differences in LOS (Kruskal-Wallis test), increasing trend in median LOS (likelihood ratio test), and Admit % across various SCS (1-5 only) (Cochran-Armitage trend test)

^d^Corresponding mean Length of stay (LOS) for each SCS in minutes (excluding visits resulting in death in ED *n* = 393)

^e^Corresponding median Length of stay (LOS) for each SCS in minutes (excluding visits resulting in death in ED *n* = 393 (1039 deaths in non-injury group))

^f^Proportion of patients admitted/transferred/died

**Table 4:** ED stay characteristics based on maximum Severity Classification Score (SCS) for non-injury visits

Total non-injury visits	ED stay characteristic	SCS	SCS^b^	SCS	SCS	SCS	SCS	Statistic^c^
		uncodable^a^	1	2	3	4	5	
		*N* = 118,716	*N* = 68,919	*N* = 73,0829	*N* = 1,097,318	*N* = 276,787	*N* = 24,688	
*N* = 2,328,916	% of all patients	5.1	3.0	31.4	47.3	12.2	1.1	---------
	LOS (mean)^d^	222.4	112.4	142.4	218.3	367.2	452.9	<0.001
	LOS (median)^e^	128	77	104	158	240	242	<0.001
	% admit/ transfer/ died %^f^	4.5	1.0	1.9	12.3	50.9	81.5	<0.001

^a^ICD-9CM code could not be mapped to SCS

^b^Severity Classification System (SCS) for all diagnoses for visit

^c^Testing differences in LOS (Kruskal-Wallis test), increasing trend in median LOS (likelihood ratio test), and Admit % across various SCS (1-5 only) (Cochran-Armitage trend test)

^d^Corresponding mean Length of stay (LOS) for each SCS in minutes (excluding visits resulting in death in ED *n* = 393)

^e^Corresponding median Length of stay (LOS) for each SCS in minutes (excluding visits resulting in death in ED *n* = 393 (1039 deaths in non-injury group))

^f^Proportion of patients admitted/transferred/died

**Table 5:** Comparison of injury to non-injury visits based on Severity Classification System (SCS)

Metric		SCS Uncodable^a^	SCS 1	SCS 2	SCS 3	SCS 4	SCS 5
% of all visits	Injury	3.8	1.2	28.6	56.0	9.6	0.8
	Non-injury	5.1	3.0	31.4	47.3	12.2	1.1
	Overall Chi-square for injury vs non-injury <0.0001						
ED LOS (mean)	Injury	191.3	109.8	138.3	170.9	284.8	386.6
	Non-injury	222.4	112.4	142.4	218.3	367.2	452.9
ED LOS (median)	Injury	123	75	112	121	180	180
	Non-injury	128	77	104	158	240	240
	Difference in median LOS between injury and non-injury visits	5.0 (3.3, 6.7)	2.0 (-0.2, 4.2)	8.0 (-8.6, 7.4)	37.0 (36.5, 37.5)	60.0 (59.3, 60.7)	60.0 (54.0, 66.0)
Admit % [For each SCS, the % admitted or transferred)	Injury	3.3	1.1	1.5	6.7	36.3	73.4
	Non-injury	4.5	1.0	1.9	12.3	50.9	81.5
	OR (Injury vs non-injury visits)	0.57 (0.54, 0.59)	1.09 (0.88, 1.36)	0.79 (0.76, 0.82)	0.51 (0.51, 0.52)	0.55 (0.54, 0.56)	0.62 (0.58, 0.66)
Median ISS	Determinable only in injury visits	1	1	1	1	3	10

^a^ICD-9CM could not be assigned an SCS level

**Fig. 1:**
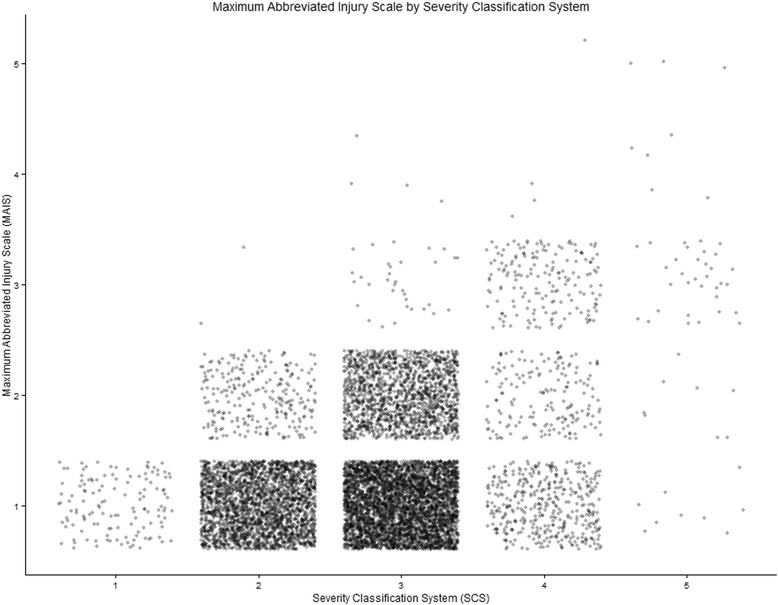
Comparison of MAIS vs SCS for the sample

Figure 1 graphically displays the frequency of MAIS versus SCS. In general, there is a slight positive association between SCS and MAIS, with MAIS being about one point lower than SCS. The majority of the sample had an SCS of 3 with an MAIS of 1 or 2, followed by an SCS of 2 with an MAIS of 1. There were more patients with an SCS 5 than all those with an MAIS of 4, 5, or 6.

## Discussion

We demonstrated that admission rates and ED length of stay increased with increasing SCS (for all visits) and MAIS severity (for injury visits). However, there was little correlation between SCS and MAIS for injury visits; increasing SCS did not consistently align with increasing MAIS for injured patients. Additionally, ED visit metrics differed between injured and non-injured patients in the same SCS categories. Specifically, injury visits had, in general, shorter lengths of stay, and were less likely to result in admission when compared to non-injury visits.

The majority of injury visit were MAIS 1 and 2, and while it would be reasonable to speculate that these visits would correspond to SCS 1 and 2, they did not. Instead, we found that although the majority of injuries were minor and could be considered not ‘clinically significant’ (ie, less than MAIS 2), these children still had moderate anticipated resource needs based on SCS scores of 2, 3, or 4. Therefore, these seemingly ‘minor’ injuries still have an important impact on healthcare utilization and ED resources. One example might be a patient who sustains an MAIS 1 laceration that necessitates repair (although this could vary from a very minor laceration easily closed with tissue glue to a small but complicated animal bite on the face with avulsion and requiring complex suturing). In some of these cases, patients may have a longer ED LOS resulting from potentially being assigned a lower triage acuity and/or requiring procedural sedation (including *nil per os* time, calling a consultant, the procedure, and recovery).

AIS and SCS are two different schemas that can be used to classify ED visits for injury that have important but unique value. (Association for the Advancement of Automotive Medicine 2008; Alessandrini et al. 2012) AIS was originally designed to compare severity of various injuries, while SCS was created as a way to compare all ED visits based on anticipated ED resource utilization. When considering categorization of emergency department injury visits, investigators using administrative datasets should select the schema that most closely aligns with their aims (i.e., injury severity versus resource utilization), or use a combined approach that incorporates both measures. Similarly, the differences in visit metrics for injured vs non-injured patients of the same SCS category should also be considered in future analyses utilizing SCS to describe anticipated resource utilization for ED visits among children. Injured patients had 35-60 min shorter lengths of stay and 5-11 % lower admission rates than non-injured patients for SCS categories 3-5. This may reflect the fact that most injured patients are not diagnostic dilemmas, and that more severely ill patients with medical illness may have increased ED LOS due to waiting for an inpatient bed. Investigators should be aware when using the SCS to compare injured and non-injured patients that their visit characteristics can differ.

The AIS has evolved over time, and many diagnoses in the current manual have severity scores that have either decreased or increased from prior versions. (Association for the Advancement of Automotive Medicine 2008) AIS was originally created as a threat to life scale, but over time has accounted for non-fatal injury morbidity. Given that many AIS 1 injuries in this dataset corresponded to an SCS 3, future versions of the AIS could consider incorporating/accounting for ED resource utilization in addition to injury severity, which could enhance the utility of AIS by using a more global definition of injury burden.

For injured patients, there was a significantly higher proportion of admitted patients with SCS 3 when compared to SCS 2, and a significantly longer LOS for SCS 4 patients when compared to SCS 3 patients, which both demonstrate face validity that the categories correspond to actual ED resource utilization in terms of ED LOS and need for admission. Similar trends were seen with increasing AIS score, although there was decrease in LOS and lower percent of patients admitted for those with MAIS 6. This is likely due to a higher proportion of patients who died in the ED, and/or the smaller sample size in this group.

This analysis has limitations that should be considered. First, since it was not a population-based cohort and most of the ED visits in the database were at pediatrics EDs in children’s hospitals, we were not able to sample children who sought care at other settings. Similarly, measures such as LOS and resource utilization may have institutional and seasonal variability. However, even though this cohort likely had more severely injured children than the general population of injured children seeking ED care across the United States, the majority of the injuries were minor. Another limitation was that we were not able to reliably identify procedures completed during the visit, which could have been related to proxies of resource utilization, including length of stay. Finally, there were visits that could not be mapped to AIS and/or SCS categories, which could bias the results. However, since this mapping was based on ICD-9-CM codes, and the more general/vague injury codes are the most likely to be unmappable, many of these injuries would have mapped to the lowest AIS and SCS categories.

## Conclusion

While admission rates and length of stay increase with increasing AIS and SCS severity, these two classification schemas do not reliably correlate with each other. Similarly, ED visit metrics differ between injured and non-injured patients in the same SCS categories. Investigators conducting analyses of administrative datasets to understand resource utilization of the injured should consider these differences carefully when describing severity for injured patients, and comparing their resource utilization to non-injured patients.
